# Functional significance of CD105-positive cells in papillary renal cell carcinoma

**DOI:** 10.1186/s12885-016-2985-7

**Published:** 2017-01-05

**Authors:** Damian Matak, Klaudia K. Brodaczewska, Cezary Szczylik, Irena Koch, Adam Myszczyszyn, Monika Lipiec, Slawomir Lewicki, Lukasz Szymanski, Robert Zdanowski, Anna M. Czarnecka

**Affiliations:** 1 Department of Oncology with Laboratory of Molecular Oncology, Military Institute of Medicine, Szaserow 128, 04-141, Warsaw, Poland; 2 School of Molecular Medicine, Medical University of Warsaw, Warsaw, Poland; 3 Department of Pathomorphology, Institute of Mother and Child, Warsaw, Poland; 4 Department of Molecular Biology, Institute of Biochemistry, Faculty of Biology, University of Warsaw, Warsaw, Poland; 5 Current address: Max-Delbrück-Centrum für Molekulare Medizin, Berlin, Germany; 6 Faculty of Pharmacy with Laboratory Medicine Division, Medical University of Warsaw, Warsaw, Poland; 7 Department of Regenerative Medicine, Military Institute of Hygiene and Epidemiology, Warsaw, Poland; 8 Institute of Genetics and Biotechnology, Faculty of Biology, University of Warsaw, Warsaw, Poland; 9 Military Institute of Hygiene and Epidemiology, Warsaw, Poland

**Keywords:** Renal cell cancer, Papillary cancer, Cancer stem cells, Tumor initiating cells, CD105, Endoglin

## Abstract

**Background:**

CD105 was postulated as a renal cell carcinoma (RCC) stem cell marker, and CD133 as a putative RCC progenitor. Hypoxia, a natural microenvironment that prevails in tumors, was also incorporated into the study, especially in terms of the promotion of hypothetical stem-like cell properties.

**Methods:**

Within this study, we verify the existence of CD105+ and CD133+ populations in selected papillary subtype RCC (pRCC) cell lines. Both populations were analyzed for correlation with stem-like cell properties, such as stemness gene expression, and sphere and colony formation. For the preliminary analysis, several RCC cell lines were chosen (786-O, SMKT-R2, Caki-2, 796-P, ACHN, RCC6) and the control was human kidney cancer stem cells (HKCSC) and renal cells of embryonic origin (ASE-5063). Four cell lines were chosen for further investigation: Caki-2 (one of the highest numbers of CD105+ cells; primary origin), ACHN (a low number of CD105+ cells; metastatic origin), HKCSC (putative positive control), and ASE-5063 (additional control).

**Results:**

In 769-P and RCC6, we could not detect a CD105+ population. Hypoxia variously affects pRCC cell growth, and mainly diminishes the stem-like properties of cells. Furthermore, we could not observe the correlation of *CD105* and/or *CD133* expression with the enhancement of stem-like properties.

**Conclusions:**

Based on this analysis, CD105/CD133 cannot be validated as cancer stem cell markers of pRCC cell lines.

## Background

Renal cell carcinoma (RCC) is the seventh most common tumor and is associated with high mortality [1]. Several subtypes of RCC have been defined: clear cell RCC (ccRCC; 70% incidence), papillary RCC (pRCC; 10% incidence), chromophobe RCC (ChRCC; 5% incidence), and rare types of RCC where the frequency is less than 1% of each [2]. The estimated statistics for 2016 in the United States predict about 62,700 new cases of kidney cancer (63.2% in men) and 14,240 deaths (64.9% in men) from the disease. The renal cancer 5-year survival rate is stage dependent and ranges from 8 to 81% for TNM stage IV and I, respectively [3]. Up to 30% of RCC patients have metastatic spread at the initial presentation [4]. Moreover, even after a nephrectomy, RCC recurs within the first 5 years in 40% of patients with an initially localized disease. As recently shown, disease recurrence and metastasis development may be mediated by cancer stem cells (CSCs) [5], which are a rare subpopulation of tumor cells suspected of playing a critical role in cancer progression. Despite the low numbers found, unique properties allow CSCs to mediate tumor development, growth, metastatic spread, and treatment resistance [6]. CSCs are characterized by self-renewal and multilineage differentiation potential. These cells may generate all cell subpopulations of the tumor and are characterized by the overexpression of Oct4, Sox2, and Nanog transcription factors (TFs) [7, 8], increased activity of ABC transporters and aldehyde dehydrogenase (ALDH)1, and the presentation of specific surface markers [9]. Moreover, CSCs are characterized by functional assays, including clonogenic assay, growth in non-adhesive spheroids, or the generation of in vivo serially transplantable carcinomas. Identification and isolation of CSCs from other tumor cells is still a source of debate [10], and it appears that all the above mentioned tests need to be conducted to properly recognize CSCs [11, 12].

In the case of RCC, putative CSCs markers were proposed, including CD105 [13], CXCR-4 [14], DNAJB8 [15], and ALDH [16, 17]. Moreover, some RCC CSC populations were isolated with functional approaches [18–24].

One of the well-known CSC inducers is hypoxia. Due to an uncontrolled proliferation of cancer cells and parallel inefficient tumor vascularization, a microenvironment within tumors is characterized by low oxygen tension (<1% O_2_) [25]. Tumor hypoxia drives the production and release of angiogenic factors [26] by cancer cells, and it may lead to changes in cancer cell metabolism. Multiple mechanisms induced by hypoxia, including microRNA expression [27] and the activation of HIF1-3 proteins [28], may promote CSC phenotypes; therefore, an oxygen microenvironment should be taken into consideration in CSC research.

In this study, we aimed to identify CD105+ cells in established RCC cell lines and to verify if those cells possess characteristics of CSCs as Bussolati et al. [13] showed that CD105+ cells isolated from primary RCC tumors have CSC characteristics. Using multiple methods, we evaluate if a similar population exists in established RCC cell lines, and verify the influence of oxygen in CSC induction, which could serve as a valuable tool to investigate CSC functions in RCC pathology.

## Methods

### Cell lines

Human primary RCC cell lines, HKCSC, 786-O, SMKT-R2, Caki-2, 769-P, and metastatic ACHN and RCC6, were used. RCC6 cells were a kind gift from Prof. Salem Chouaib (INSERM, Institut Gustave Roussy, Villejuif, France). SMKT-R2 cells were gifted by Prof. T. Tsukamoto and Dr. S. Tochizawa (School of Medicine, Sapporo Medical University, Sapporo, Japan). Also, 786-O, Caki-2, ACHN, and 769-P were bought from ATCC (Manassas, VA, USA), and human healthy kidney epithelial cell line ASE-5063 was bought from Applied StemCell Inc. (Milpitas, CA, USA). HKCSCs (human kidney CSCs; 36117-44-T75 of papillary origin) were obtained from Celprogen (Torrance, CA, USA). All cell lines were analyzed until the tenth passage of cells, which were originally stocked in our laboratory.

### Cell culture

Cells were cultured in 75 cm^2^ cell culture flasks (Orange Scientific) in RPMI-1640/GlutaMax medium (Gibco, Paisley, Scotland, UK), apart from HKCSCs, which were maintained in Human Kidney Cancer Stem Cell Complete Growth Medium (Celprogen, Torrance, CA, USA). RPMI-1640 was supplemented with 10% FBS (Hyclone, Utah, USA/Gibco, Paisley, Scotland, UK) and treated with antibiotics (Penicillin-Streptomycin solution, Sigma, St. Louis, MO, USA) to a final concentration of 100 IU/mL penicillin and 100 μg/mL streptomycin. After thawing, the cells were passaged at least once before being used in experiments. After reaching 80% of confluence, the cells were harvested by trypsinization (0.25% trypsin, 0.03% EDTA solution; Invitrogen, Carlsbad, CA, USA), counted in an automated cell counter MOXI Z (Orflo, Ketchum, ID, USA), and used in experiments described below. Cells were seeded at an appropriate concentration in different tissue culture vessels (Table 1) and kept in an normoxic incubator for 24 h to allow cells to attach and then be used in assays. In the hypoxia experiments, after an initial 24 h incubation in normoxia, the cells were moved into a hypoxic incubator, cultured for an appropriate amount of time, and used in cell assays. Cells cultured in normoxic conditions served as a control. Cells were cultured according to standard mammalian tissue culture protocols and sterile techniques in normoxia (5% CO_2_; 37 °C) and hypoxia (1% O_2_; 5% CO_2_; 37 °C) incubators.

**Table 1:** Number of cells seeded in culture vessels

Cell line	T75	6 well plate	96 well plate
Caki-2	500 000	60 000	1000
ACHN	950 000	120 000	2000
HKCSCs	250 000	30 000	500
ASE	1 200 000	150 000	3000

### Cell proliferation measurement

Cells were cultured in 96-well plates as described above. The measurement of cell viability using alamarBlue® (Invitrogen, Carlsbad, CA, USA) was performed every day for a total of 6 days according to the manufacturer’s protocol. The medium was exchanged after 3 days of culture. The absorbance of alamarBlue® was measured using Multiskan™ GO Microplate Spectrophotometer (Thermo Fisher Scientific, Waltham, MA, USA) at 570 nm and 600 nm. The absorbance obtained from readings was then calculated to a percentage reduction of alamarBlue®.

### Cell cycle analysis

Cells were cultured in 6-well plates as described above. After 72 h incubation, cells with approximately 80% confluence were collected with trypsin. The cells were washed in PBS and fixed in ice-cold 70% ethanol for 30 min at −20 °C. The fixed cells were washed three times and suspended in PBS containing 50 μg/mL of RNase A for 30 min, then incubated with 50 μg/mL propidium iodide (PI; Cayman Chemical, Ann Arbor, MI, USA) for 30 min in the dark. Subsequently, the samples were acquired in a FACSCalibur (Becton Dickinson, San Diego, CA, USA) system and PI incorporation was estimated using CellQuest Pro software (Olympus, Tokyo, Japan). For each measurement, at least 25,000 cells were acquired. An analysis of the cell cycle was performed using the Watson modelling algorithm from the FlowJo software (Ashland, OR, USA).

### Gene expression analysis

Cells were cultured in 75 cm^2^ cell culture flasks as described above. After 72 h incubation, cells with approximately 80% confluence were collected by trypsinization. Total RNA Mini Plus (A&A Biotechnology, Gdynia, Poland) was used for total RNA isolation according to the manufacturer’s protocol. Eventually, RNA was eluted from the column with RNase-free water. The concentration and purity of RNA were determined by measuring the absorption at 230 nm, 260 nm, and 280 nm in a Multiskan™ GO Microplate Spectrophotometer. A TranScriba Kit (A&A Biotechnology, Gdynia, Poland) was used to generate full-length first strand cDNA from total RNA templates. An oligo-(dT) primer was used for the transcription of RNA by a Moloney murine leukemia virus reverse transcriptase. Five μg of total RNA in 20 μL of reaction mix was used for reverse transcription. Single strand complementary DNA (cDNA) generated in rtPCR was used as a template for amplification by sqPCR. Real-time PCR was performed with gene-specific primers and probes (listed in Table 2) and TaqMan® Universal Master Mix II, no UNG (Applied Biosystems, Foster City, CA, USA). Reactions were performed in 20 uL volume, with 2 μL of cDNA template, according to the manufacturer’s instructions, on the StepOne real-time PCR system (Applied Biosystems, Foster City, CA, USA) in 48-well plates. Data were calculated with the 2^(−Delta C(T))^ method, with normalization to mean expression of *peptidylprolyl isomerase A* (*PPIA*) as a housekeeping gene.

**Table 2:** Primers used for real time PCR

Gene	Assay ID
*hif1*	Hs00153153_m1
*hif2*	Hs01026149_m1
*ppia*	Hs01565700_g1
*oct4*	Hs04260367_gH
*sox2*	Hs01053049_s1
*cd105*	Hs00923996_m1
*cd133*	Hs01009257_m1

### Fluorescence-activated cell sorter

Cells were cultured in 6-well plates as described above. After 72 h culture, cells with approximately 80% confluence were collected with Accutase (BDSciences, San Diego, CA, USA) and analyzed for CD105 and CD133 marker expressions. Cells were stained with (i) CD105-PE (BD Pharmingen, San Diego, CA, USA, clone 266) plus CD133/1-APC (Miltenyi Biotec, Bergisch Gladbach, Germany, clone AC133); or (ii) CD105-FITC (Biolegend, San Diego, CA, USA, clone 43A3) plus CD133/2-APC (Miltenyi Biotec, Bergisch Gladbach, Germany, clone 293C3) according to the manufacturers’ protocols with the appropriate isotype controls. Subsequently, the samples were acquired in a FACSCalibur system. For each measurement, at least 10,000 cells were acquired. Data were analyzed by Flowing Software (Turku Centre for Biotechnology, Turku, Finland).

### Colony formation assay

The ability to create colonies was measured with the use of the semi-soft agar method [29]. Cells after passage were collected as described above, and 2,000 cells were seeded into a 96-well plate according to the Stem Cell Colony Formation Assay (Cell Biolabs, San Diego, CA, USA) protocol. Cells were cultured for 21 days, and every 7 days photos of the colonies were taken using an inverted light microscope (Olympus CKX41, Tokyo, Japan) with CellSens software (Olympus, Tokyo, Japan). Additionally, colonies formed after 2 weeks were used for quantitative analysis; agar was liquefied by Lysis Solution and repetitive pipetting. Half of the obtained colonies were mixed 1:1 with the RPMI medium containing 20% alamarBlue® in a 96-well plate and after 3 h incubation they were analyzed as described in Section 2.4.

### Sphere formation assay

After passage, cells were collected as described above and washed twice with PBS to remove any remaining medium with serum. Cells were counted and seeded at 5,000 cells/mL on non-adherent 24-well plates (TC plate, suspension, F, Sarstetd, Numbrecht, Germany) in 500 mL per well of DMEM (with GlutaMax, Gibco, Paisley, Scotland, UK) and supplemented with antibiotics (100 IU/mL penicillin and 100 μg/mL streptomycin, Sigma, St. Louis, MO, USA), b27 (1×; Gibco, Paisley, Scotland, UK), epidermal growth factor (20 ng/mL; Invitrogen, Carlsbad, CA, USA), and basic fibroblast growth factor (25 ng/mL; Invitrogen, Carlsbad, CA, USA). Cells were cultured for 5 days and photos of the spheroids were taken using an inverted light microscope (Olympus CKX41, Tokyo, Japan). For quantitative analysis, formed spheroids were counted from five representative microscope fields by two independent experimenters using CellSens software (Olympus, Tokyo, Japan).

### Wound healing assay

Cell migration was determined using a wound healing assay. After passage, cells were collected as described above and seeded in 6-well plates, in an appropriate medium, in densities forming a confluent monolayer. After 24 h incubation, a linear wound was created using a 1 mL pipette tip. Cells were washed with PBS to remove detached cells and were cultured in an appropriate medium for 24 h in hypoxic and normoxic incubators. The wound spaces were imaged under an inverted microscope (at 40× magnification; Olympus) at 0 h and 24 h at the same field. Wound healing was analyzed using WimScratch software, according to the following formula: wound healing area (%) = [cell-free area (0 h) − cell-free area (24 h)]/cell-free area (0 h) × 100. Each scratch was performed in triplicate.

### Hanging drop assay

After passage, the cells were collected as described above and seeded at 500 cells per 15 μL drop of appropriate medium on the inner side of a 100 mm Petri dish lid. The lid was turned upside down and placed on top of the dish filled with 10 mL of PBS. Cells were observed daily for 10 days to detect the creation of aggregates. Photos were taken using an inverted light microscope (Olympus CKX41, Tokyo, Japan). For quantitative analysis, areas of formed aggregates were counted from three representative microscope fields (drops) by two independent experimenters using CellSens software (Olympus, Tokyo, Japan).

### Immunocytochemistry

Cells were cultured in 75 cm^2^ cell culture flasks as described above. After 72 h incubation, cells with approximately 80% confluence were collected with Accutase, and suspended in PBS. Then cells were centrifuged and re-suspended in 4% PFA for 10 min at room temperature. PFA was removed by centrifugation, and cells were placed in ddH_2_O onto ICC SuperFrost microscope slides (Thermo Scientific, Hennigsdorf, Germany, 10143560WCUT) and allowed to dry. The staining was performed in a Flex Autostainer instrument (Dako, Glostrup, Denmark) and with the use of EnVisionFlex kits (Dako, Glostrup, Denmark). Antigen retrieval was performed with 5 min proteinase incubation for CD105 or 1 h incubation in a target retrieval solution (pH = 6) for CD133. After the blockade of endogenous peroxidase activity, the slides were incubated for 1 h with primary antibodies: mouse anti-human CD105 (Dako, Glostrup, Denmark, M3527, clone SN6h), or rabbit anti-human CD133 (Biorbyt, Cambridge, UK, polyclonal, orb18124). After washing, the slides were incubated with appropriate secondary antibodies labelled with HRP. For CD105 staining, HRP activity was amplified with the FLEX+ Mouse Linker. Visualization was performed with Flex DAB+ chromogen. Slides were counterstained with hematoxylin (Dako, Glostrup, Denmark, CS700), coverslipped with Pertex (HistoLab, Gothenburg, Sweden, 00840) and observed in a light microscope (Olympus CKX41, Tokyo, Japan).

### Statistical analysis

Data are presented as the mean values ± SD or ratios (hypoxia/normoxia). Each experiment was performed at least three times with three technical replicates. The differences between the groups studied were considered significant when the *P*-value was less than 0.05 in the Student’s *t*-Test calculated in MS Excel (Microsoft), and were designated with an asterisk on graphs (*P* < 0.1 is designated with a double asterisk).

## Results

### CD105+ and CD133+ subpopulations analysis in RCC cell lines

**Fig. 1:**
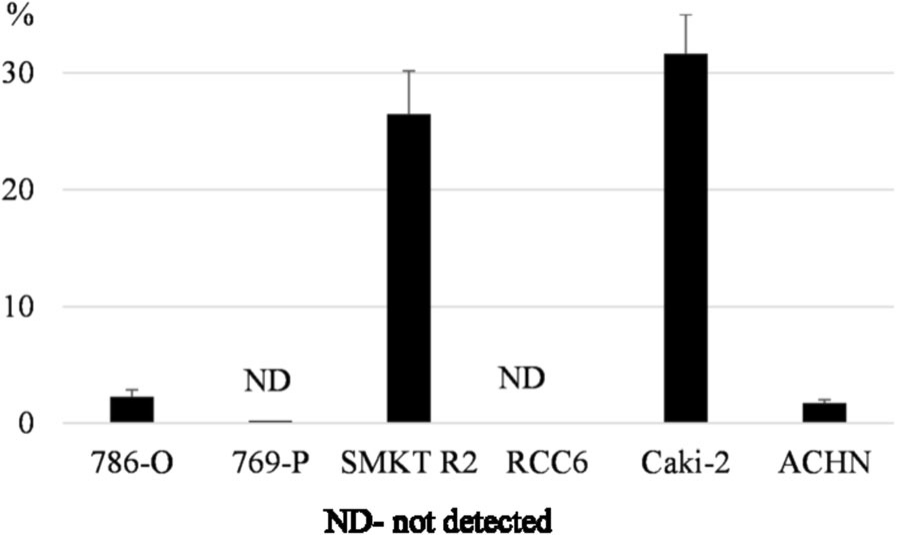
Percentage of CD105 positive cells within RCC cell lines. RCC cell lines were cultured in normoxic conditions, and after the third day, cells were analyzed by flow cytometry for the CD105 surface marker. The graph shows a relative amount of CD105+ cells in relation to isotype control (threshold). The highest number of CD105+ (more than 25%) was identified in the primary tumor derived Caki-2 and SMKT-R2 cell lines. Similar CD105+ levels were observed in another primary tumor derived 786-O and metastatic ACHN, while in 769-P (primary) and RCC6 (metastatic) no positive cells were detected

**Fig. 2:**
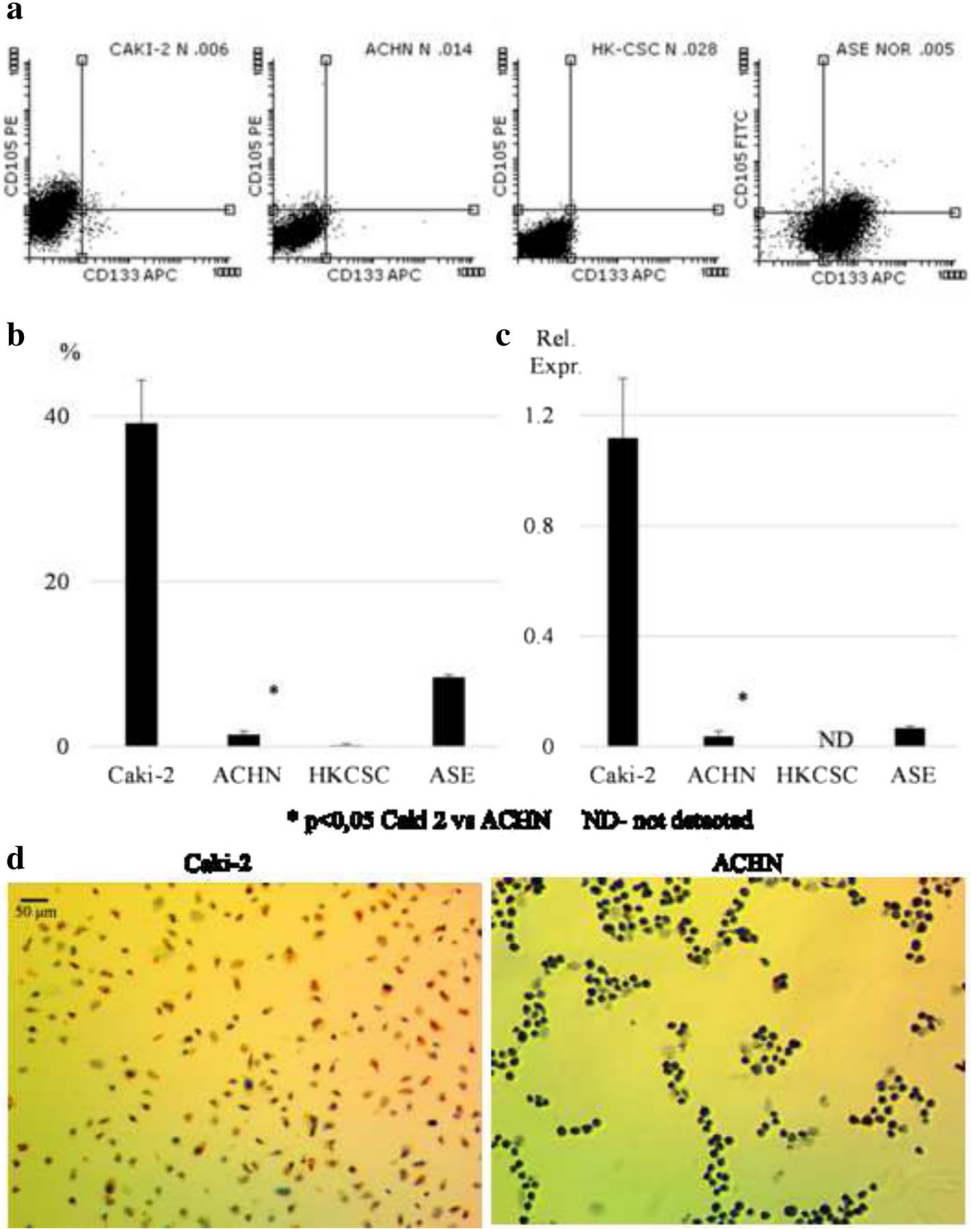
CD105 expression on protein and mRNA level. For further analysis, Caki-2 (high expression), ACHN (low expression), HKCSC (control), and ASE (control) were used. **a** Representative dot plots of CD105 and CD133 expression in tested cell lines. **b** Percentage of CD105+ cells in tested cell lines measured by flow cytometry. Within control cell lines, only normal renal cells of embryonic origin (ASE) had a CD105+ population, while in the commercially available renal cancer stem cell line (HKCSC), this population was hardly detected. **c** Relative expression of *CD105* gene was measured by real-time PCR in relation to the *PPIA* housekeeping gene. *CD105* expression was significantly upregulated in Caki-2 and downregulated in ACHN; a similar observation was made in the FACS analysis. **d** ICC staining was done to confirm Caki-2 and ACHN flow cytometry results. Around one-third of Caki-2 cells were positive for the CD105 marker with significant expression. However, in ACHN CD105+ cells were not detected with this method

Among an evaluated panel of established RCC cell lines, including primary tumor derived and metastasis derived (Table 3), the CD105+ subpopulation was confirmed with FACS in majority of cell lines with Caki-2 and SMKT-R2 cell lines presenting the highest number of positive cells, which was more than 25%. In the 786-O and ACHN lines, less than 5% of cells were CD105+, while no positive cells were detected in 769-P and RCC6 (Fig. 1). As a positive control, HKCSCs obtained from a primary papillary RCC tumor [30] were used, but the CD105+ subpopulation was hardly detectable in this cell line. At the same time, a substantial number of CD105+ cells were detected in an ASE cell line, which were normal renal cells of embryonic origin (Fig. 2a and b).

**Table 3:** Summary of cell line origin and CD105/CD133 occurrence

Cell line	RCC subtype (reference)	Stage	CD105		CD133	
			FACS	Real-time PCR	FACS	Real-time PCR
786-O	clear cell [42–44]	primary	+	NA	NA	NA
769-P	clear cell [42, 43]	primary	ND	NA	NA	NA
SMKT-R2	Mixed [45]	primary	+++	NA	NA	NA
RCC6	Papillary [46]	primary	ND	NA	NA	NA
Caki-2	Papillary [31, 32, 47]	primary	+++	+++	+	+++
ACHN	Papillary [32, 48]	metastatic	+	+	+	ND
ASE	Normal^a^	embryonal	++	+	+++	++
HKCSC	Papillary^a^	primary	+/ND	ND	+/ND	ND

+++ - strong expression, ++ - mean expression, + - weak expression

*ND* not detected (no expression), *NA* not applicable; not determined

^a^Supplier Certificate of Analysis

For further analyses, HKCSCs, ASE, Caki-2 (high CD105 expression), and ACHN (low expression) cell lines were selected. Caki-2 and ACHN cell lines were recently evaluated as derivatives of papillary RCC [31–34]; therefore, we have focused on these cell lines because CSCs in pRCC have not been described until today.

A high number of CD105+ cells in Caki-2 were confirmed in ICC staining—one-third of the cells were positive for this marker (Fig. 2d)—and CD105 expression was detected on the mRNA level (Fig. 2c). In contrast, CD105+ cells in ACHN could not be detected in the ICC method (Fig. 2d), but low expression of this gene was found by the qPCR approach (Fig. 2c).

**Fig. 3:**
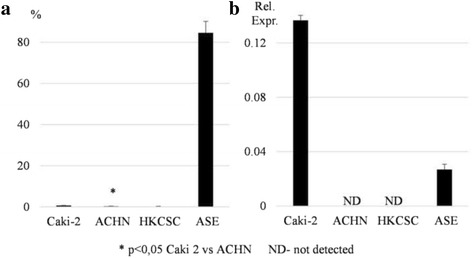
CD133 expression on protein and mRNA levels. The CD133 receptor was evaluated within CAKI-2, ACHN, HKCSC, and ASE cell lines. **a** Percentage of CD133+ cells measured by flow cytometry. Caki-2 had a significantly higher number of CD133+ cells than ACHN. An extremely high number of CD133+ population was identified in ASE; in contrast, in HKCSC, the population was not detected. **b** The relative expression of *cd133* measured by real-time PCR normalized to the *PPIA* housekeeping gene. Gene expression showed a different profile in comparison to flow cytometry; the relative expression of *cd133* was higher in Caki-2 than ASE. The gene expression was not detectable in ACHN and HKCSC

The CD133 receptor as the RCC progenitor cell’s putative marker [35–37] was also evaluated. The Caki-2 cell line had a slightly larger CD133+ subpopulation than the ACHN cell line (Fig. 3a), but mRNA was detectable only in the former (Fig. 3b). The number of CD133+ cells in both cell lines was very low as established by FACS and ICC (data not shown) consistently with previously published data for RCC cell lines [38]. Interestingly, CD133 expression was significant in the ASE cell line as most cells were positive for this marker. This was also consistent with data reported elsewhere for both fetal [39] and adult renal cells [40].

### CD105 populations and stem-like properties

**Fig. 4:**
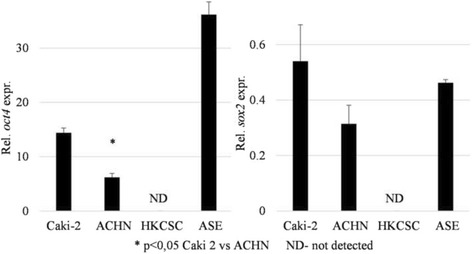
Expression of “stemness genes”. Relative expression of *oct4* and *sox2* stem-related genes was measured by real-time PCR in relation to the *PPIA* housekeeping gene. Statistically significant differences were obtained between Caki-2 and ACHN for the *oct4* gene. Interestingly, in HKCSC, neither gene was detected. In contrast, the *oct4* relative expression in ASE (normal renal cells of embryonic origin) was significantly higher than in the other cell lines

To test if the CD105 expression correlated with the stem-like properties of pRCC cell lines, the expression of *oct4* and *sox2* genes—markers of embryonic, adult, and CSC—was evaluated, but only *oct4* expression was significantly higher in CD105-high cells (Caki-2). The expression of *oct4* and *sox2* was also detected in ASE cells, but not in HKCSCs (Fig. 4).

**Fig. 5:**
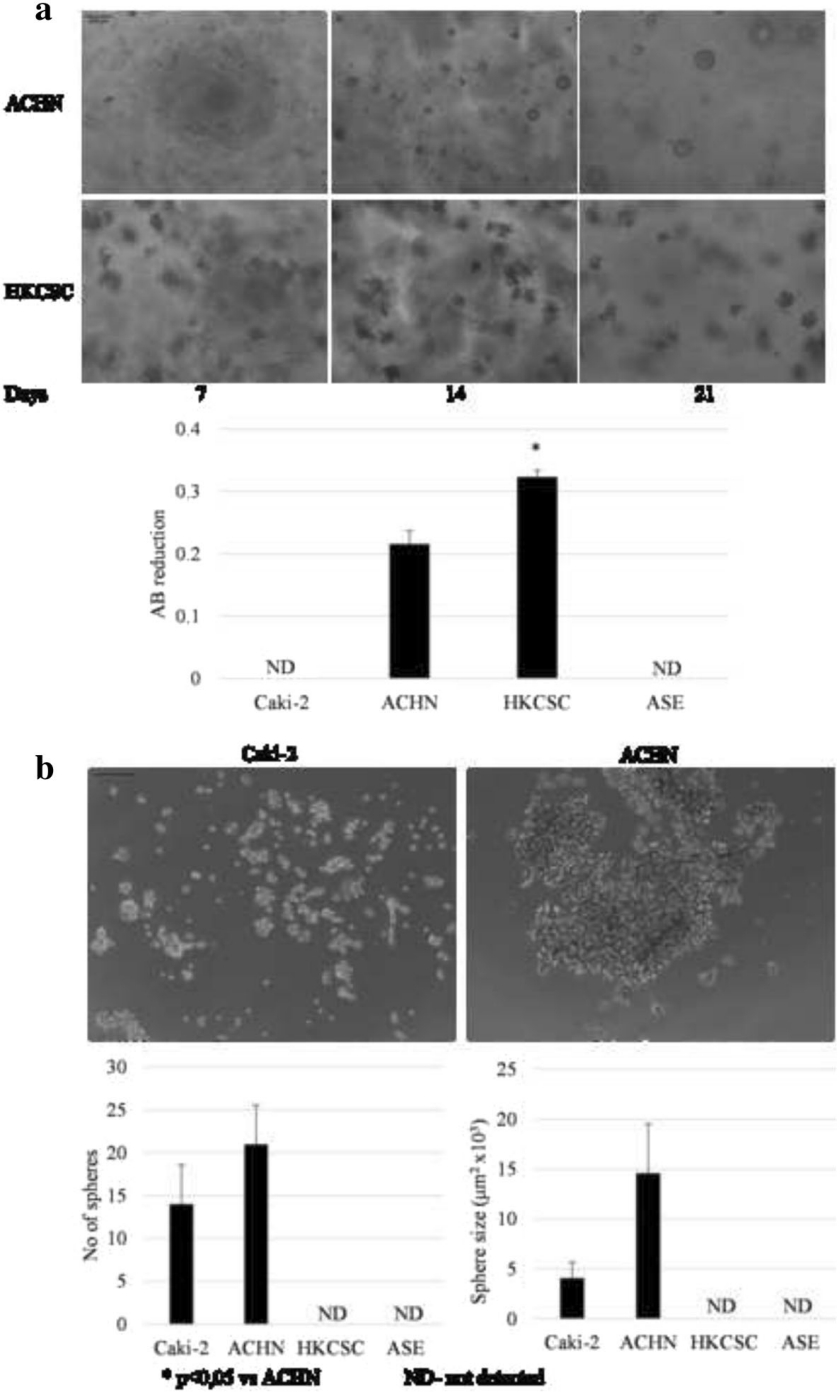
Colony- and sphere-forming abilities of pRCC cell lines. **a** Representative images of colonies formed in semi-soft agar at different time points of culture. From four tested cell lines, only two (ACHN and HKCSC) were able to generate colonies in semi-soft agar. The differences in colony morphologies were noticed: ACHN created round-shaped colonies and HKCSC irregular-shaped. A quantitative analysis of viable colonies was done using the alamarBlue® assay: HKCSCs created significantly more colonies than ACHN. **b** Representative images of cells grown in sphere-promoting conditions on the fifth day of culture. ACHN were able to create aggregate-like structures. Caki-2 spheres were smaller and more round-shaped. The other cell lines, HKCSC and ASE, did not form spheres in these conditions. A quantitative analysis of Caki-2 and ACHN spheres number showed no significant differences, although the ACHN spheres were significantly bigger than the Caki-2 spheres

Sphere and colony formation assays [41] were used for the functional identification of stem-like cell-rich cultures. Only ACHN and HKCSCs were able to form colonies in semi-soft agar (Fig. 5a). A quantitative AlamarBlue assay showed a significantly increased amount of viable HKCSC colonies compared with ACHN. After 2 weeks of culture, ACHN cells created round, sphere-like colonies that increased in volume until the third week. HKCSCs formed mulberry-like colonies with rough edges as soon as 7 days after seeding; however, the colonies seemed not to grow significantly during further culture.

In sphere-promoting culture conditions (DMEM, FGF, EGF, and B27 medium), only ACHN and Caki-2 cells were viable (Fig. 5b). ACHN cells created large aggregates that fused over time, while Caki-2 cells formed smaller, irregular sphere-like structures. ACHN spheres were significantly bigger than Caki-2 spheres, although there were no significant differences in sphere number between them.

**Fig. 6:**
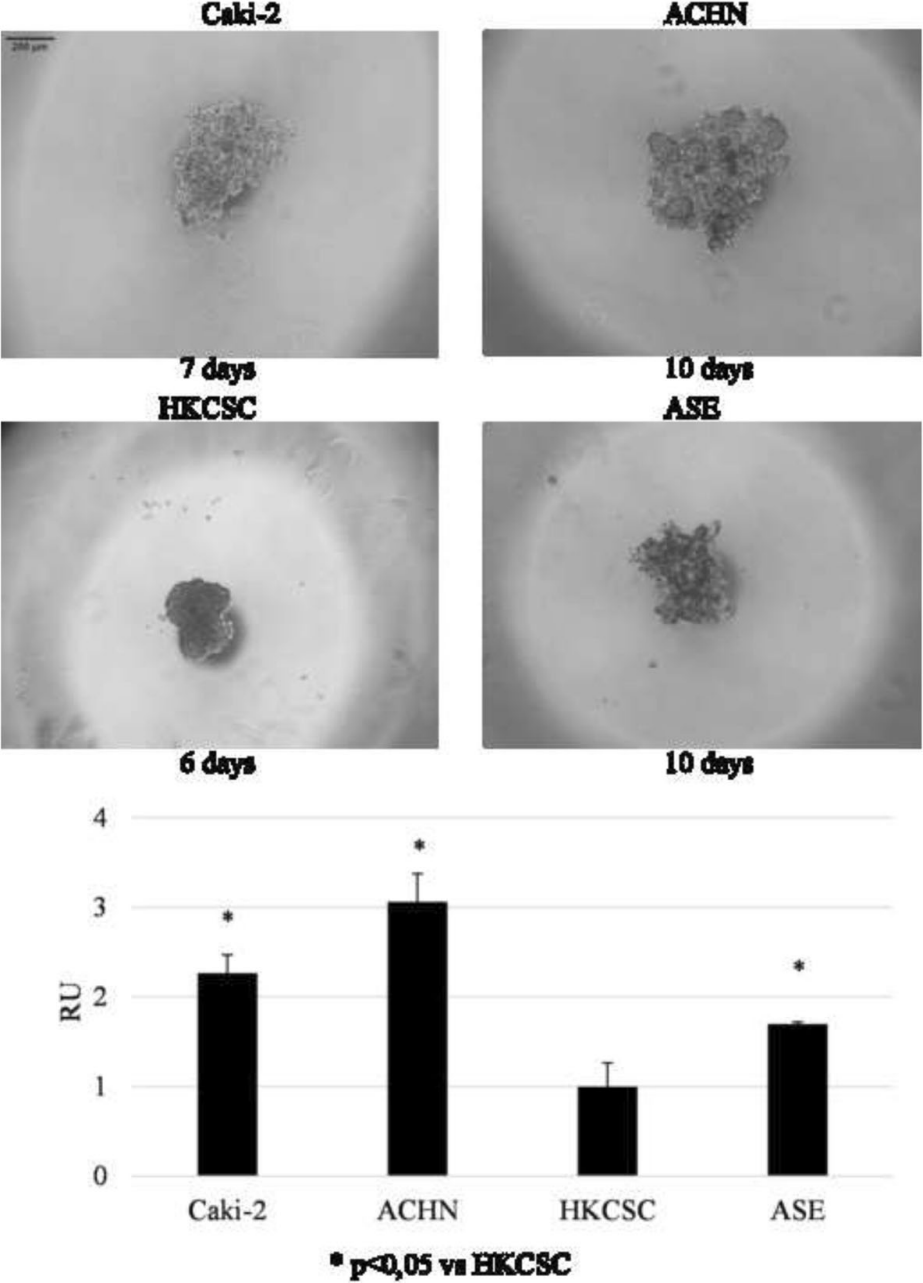
Hanging drop assay of pRCC cell lines. Representative images of aggregates formed by tested cell lines in a hanging drop assay. The HKCSC cell line generated a homogeneous, compact 3D aggregate, while Caki-2, ACHN, and ASE remained loose and were bigger than the structure of HKCSC

Cell–cell cohesion properties were screened in a hanging drop assay, and Caki-2 and ASE cells formed the aggregates, while HKSCSs created a compact structure with firm edges (Fig. 6). Throughout the study, an ACHN line grew in culture as loose cells. Less compacted structures of Caki-2, ACHN, and ASE aggregates were significantly bigger in size than the compacted HKCSC.

### Hypoxia differentially affected pRCC cell growth

**Fig. 7:**
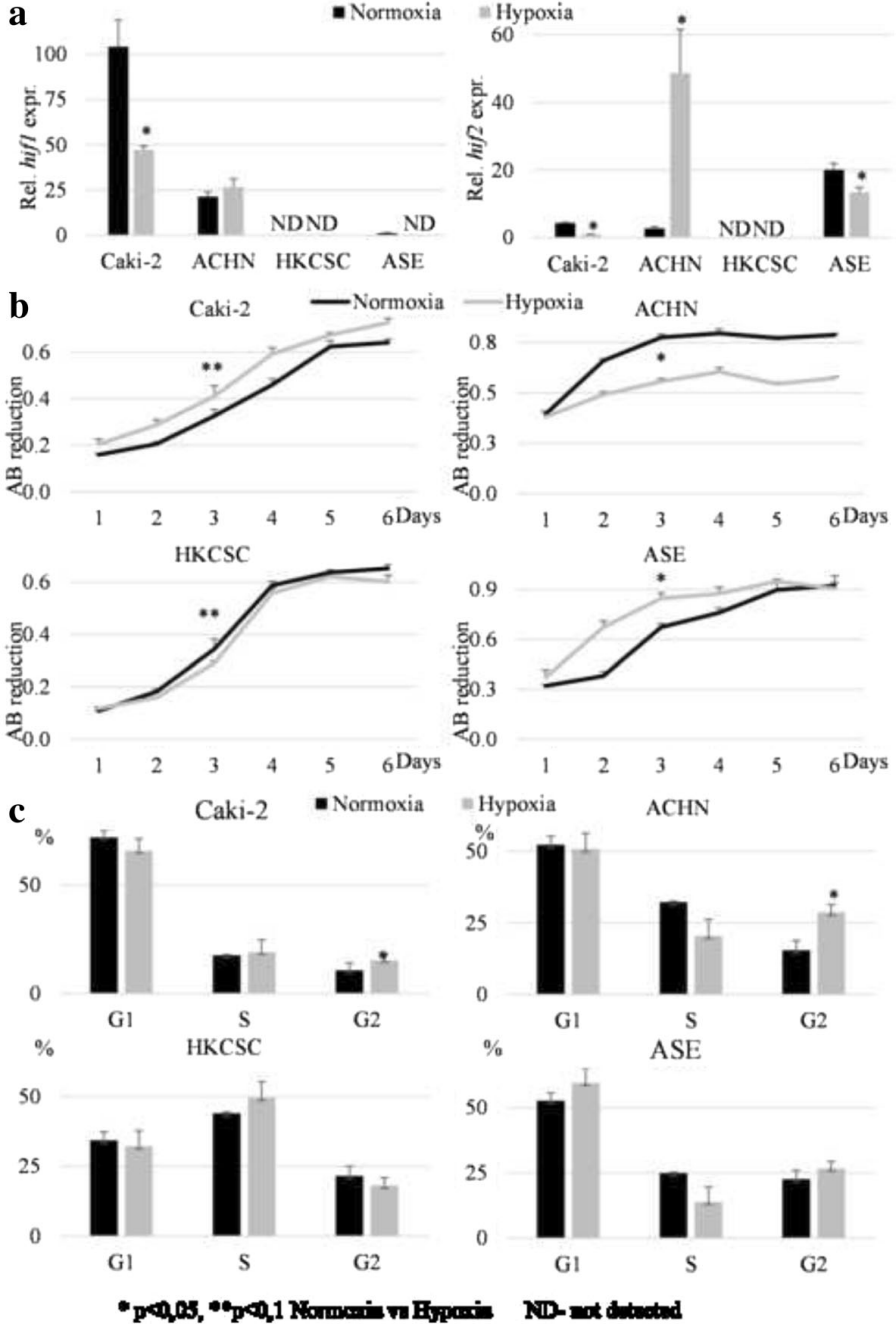
The influence of oxygen on pRCC cell growth. **a** Relative expression of *hif1* and *hif2* genes was measured in real-time PCR in relation to the *PPIA* housekeeping gene. Overexpression of *hif1* was identified in Caki-2 and ACHN at various oxygen partial pressure compared to the control line (ASE). In hypoxia, *hif1* expression was significantly downregulated in Caki-2 only. Relative expression of *hif2* showed a different profile than *hif1*; hypoxia significantly downregulated the gene expression in Caki-2 and ASE, although in ACHN, a strong upregulation was observed. In both cases, *hif1* and *hif2* were not detectable in HKCSC. **b** Growth curves of cells cultured in monolayer in normoxic and hypoxic incubators were determined with alamarBlue® assay. The third day of culture was taken to statistical analysis because of the strategy for further experiments. In the observation of overall growth trends, hypoxia promoted proliferation of Caki-2 and ASE cell lines, and inhibited the proliferation of ACHN in a parallel lack of the influence on HKCSC. **c** The percentage of cells in different phases of the cell cycle was analyzed by flow cytometry with PI staining. The analysis revealed a significant influence of hypoxia on the G2/M arrest of ACHN, which correlated with the proliferation inhibition. In the case of Caki-2 cells, which proliferated more in low O_2_, an increase in the percentage of cells in G2 phase was observed. For the rest of the cell lines, oxygen partial pressure had no effect on the cell cycle

Cell line characteristics also were described in hypoxic conditions with the assumption that low O_2_ would promote stemness features. Both pRCC cell lines Caki-2 and ACHN overexpressed *hif1*, in comparison to tested normal renal ASE cells, but the level of the *hif2* gene was lower than in the ASE cells (Fig. 7a). In response to hypoxia, ACHN cells up-regulated *hif2* expression and reduced growth by G2/M arrest (Fig. 7c). In the case of Caki-2 cells, the reaction was the opposite; the expression of both *hif1* and *hif2* was slightly decreased but the cells exhibited a mild increase in the rate of proliferation in hypoxia, as did normal renal cells (Fig. 7b).

**Fig. 8:**
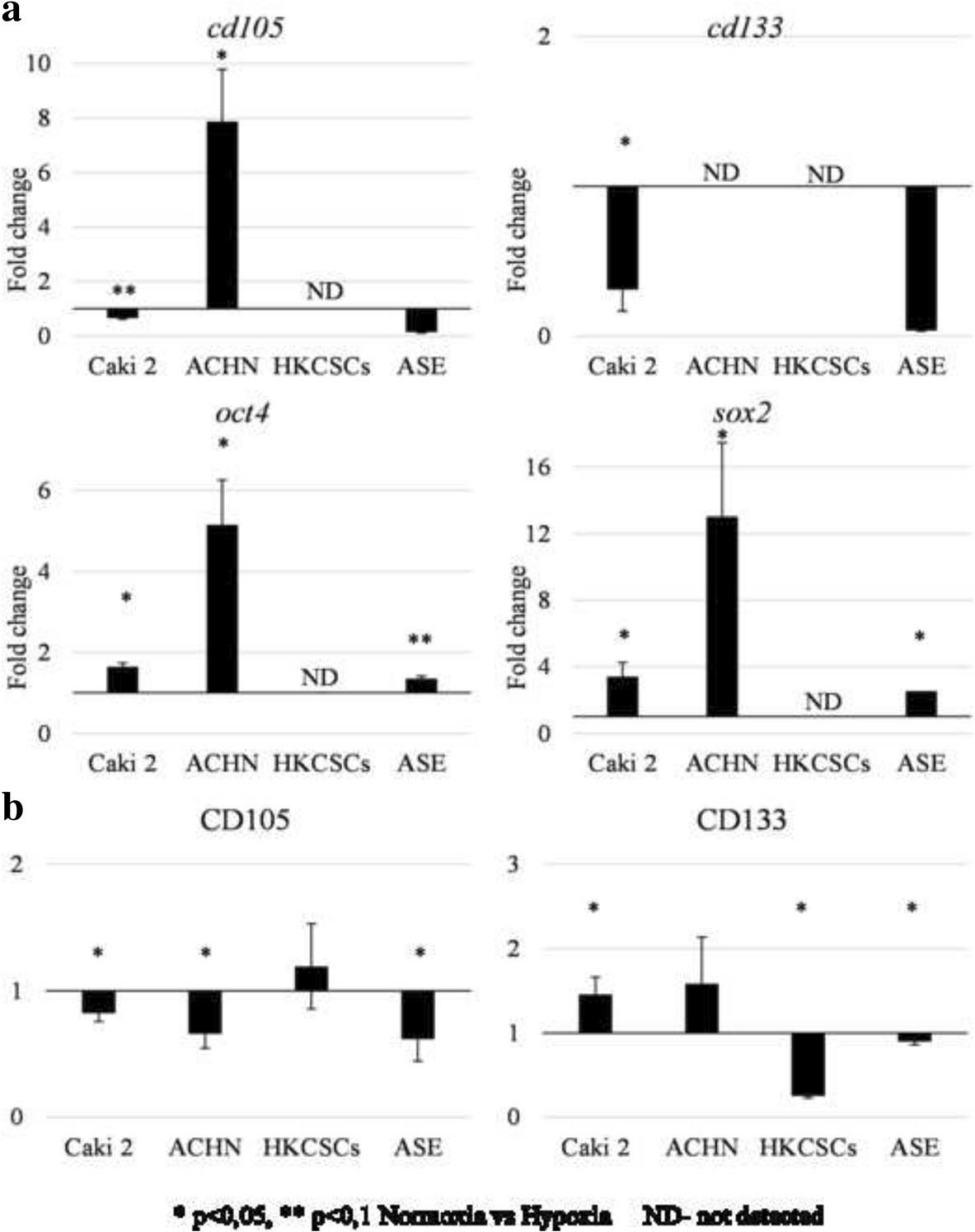
The influence of oxygen on gene expression. **a** Relative expression of *cd105*, *cd133*, *oct4*, and *sox2* was measured by real-time PCR and presented as a fold change (hypoxia/normoxia). Both Caki-2 and ACHN cell lines had an increased expression of TF genes (*oct4* and *sox2*) in hypoxia, similar to ASE. The expression of membrane marker genes (*cd105* and *cd133*) was downregulated in Caki-2 and ASE by low O_2_. The opposite phenomenon was obtained for *cd105* and ACHN: hypoxia induced overexpression. Intriguingly, in HKCSC, none of the genes were detectable. **b** The percentage of CD105+ and CD133+ cells in tested cell lines was measured by flow cytometry and presented as a fold change (hypoxia/normoxia). Results from real-time PCR were confirmed for both proteins, and a reduced amount of the CD105+ and CD133+ populations in Caki-2 and ASE were observed in hypoxia. In contrast to gene expression, the CD105+ population was reduced in ACHN hypoxic cells

In response to hypoxia, the ACHN cell line increased the expression of stemness TFs: *oct4* and *sox2* (Fig. 8a). The CD133+ subpopulation enumeration was slightly increased by the low level of O_2_ (Fig. 8b), but the percentage of CD105+ cells slightly reduced with the increase in CD105 mRNA levels. Likewise, the Caki-2 cell line reaction to hypoxia was similar: stem-related TFs and CD133 expression (the latter at the protein level only) increased, but CD105 decreased (Fig. 8). Conversely, in the ASE cell line, both CD133 and CD105 subpopulations reduced in hypoxia. However, *oct4* and *sox2* expression increased in low O_2_. This shows the differential effect of oxygen levels on pRCC cell line features.

**Fig. 9:**
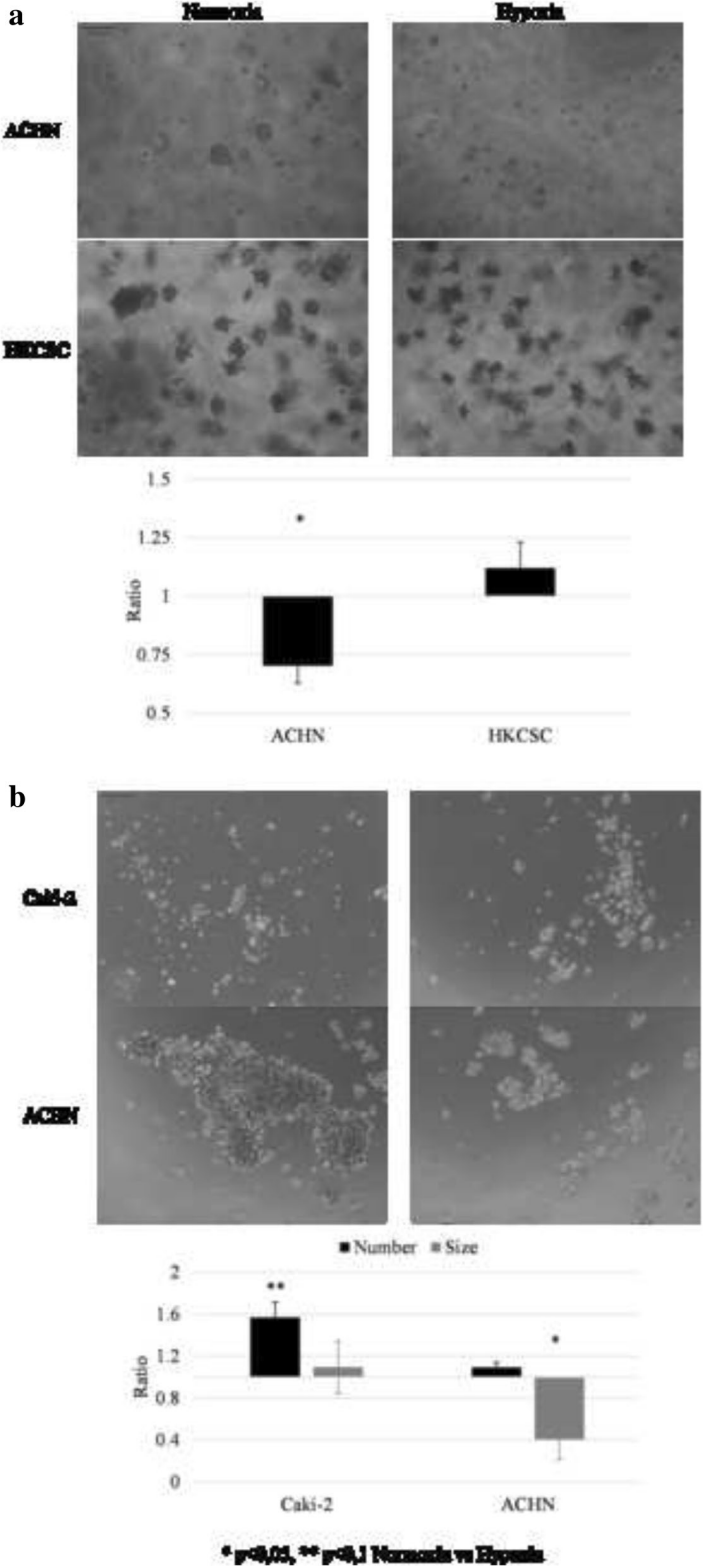
The influence of oxygen on colony- and sphere-forming abilities. **a** Representative images of colonies were formed in semi-soft agar in normoxic and hypoxic cultures. The number of viable ACHN colonies in normoxia was significantly bigger than in hypoxia, while there were no differences in HKCSC. **b** Representative images of cells grown in sphere-promoting conditions at the fifth day of culture in normoxia and hypoxia. The ACHN cell line showed reduced colonies and spheres in hypoxia, which is a reflection of similar hypoxic inhibition in monolayer culture. Caki-2 was not able to create colonies; nevertheless, its number of spheres was greater in hypoxia, as was hypoxia-promoted Caki-2 monolayer proliferation. Alternatively, HKCSC created colonies but not spheres

Despite the induction of the expression of some stem-related genes by hypoxia, stem phenotype is mostly not expressed more intensively in low O_2_ in tested pRCC cells (Fig. 9). ACHN cells showed reduced colony- and sphere-forming abilities in hypoxia along with reduced cell growth (alamarBlue® assay; Fig. 7b). At the same time, HKCSCs, which grow equally well in hypoxia and normoxia in 2D, showed a different morphology of colonies created in semi-soft agar. The hypoxia colonies were smaller and less compacted than the normoxic ones. Only Caki-2 cells developed more spheres in low O_2_ compared to spheres developed in normoxia.

**Fig. 10:**
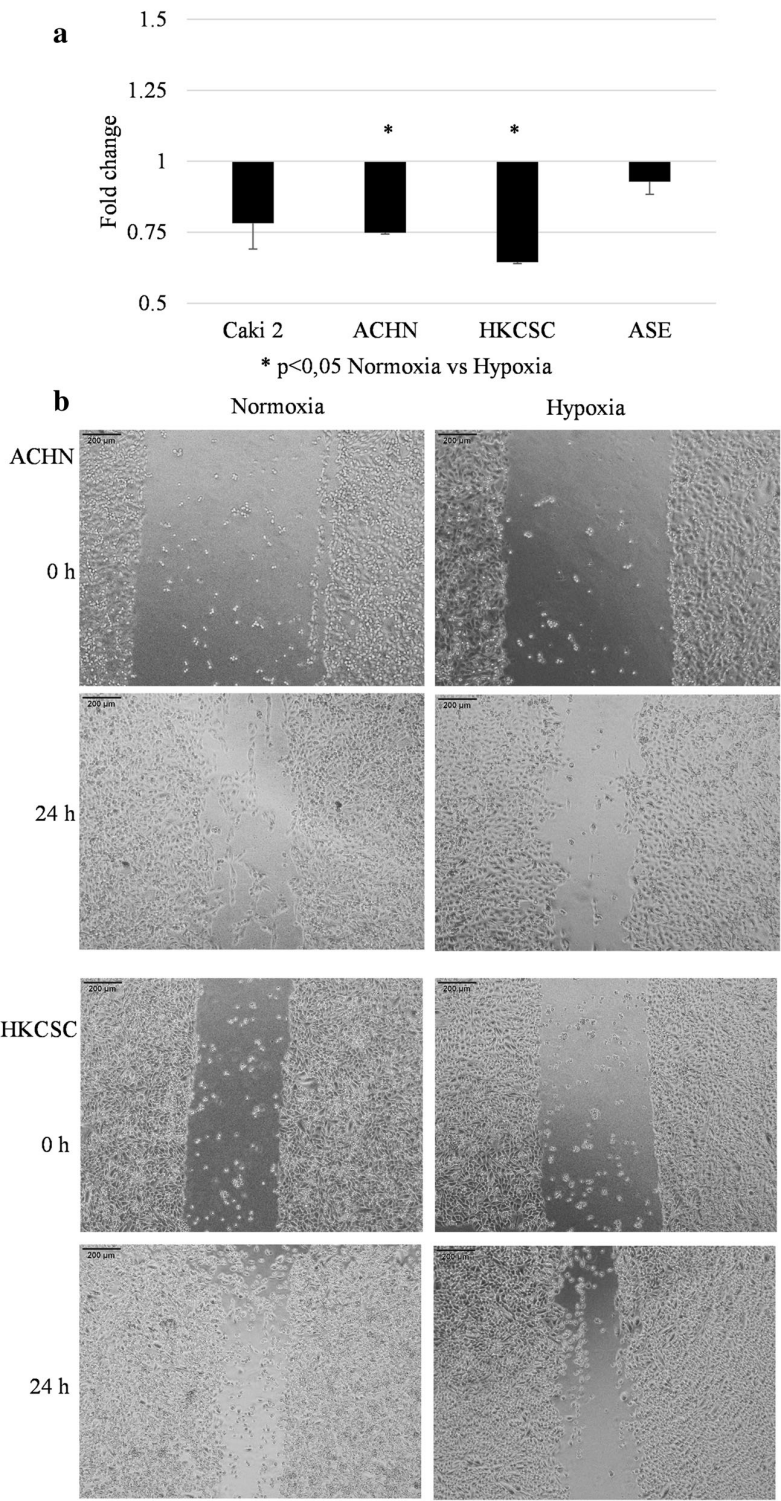
Wound healing assay. **a** ACHN and HKCSC cell lines relatively covered scratched in hypoxia significantly less than in normoxia; hypoxia inhibited the cell migration potential of those cells. However, in Caki-2 and ASE migration, potential differences in terms of oxygen were not significant. **b** Representative images of migrating ACHN and HKCSC in normoxic and hypoxic conditions at 0 h and 24 h

The wound healing assay showed the most reduced cell migration ability of all the tested cell lines in hypoxia (Fig. 10a). However, the hypoxia inhibition of cell migration was statistically significant only in ACHN and HKCSC. Photos were taken immediately after scratch and after 24 h hypoxic or normoxic incubation (Fig. 10b). This indicated that the migration capacity of the analyzed cell lines was inhibited by low O_2_.

### Comparison of the cell line origin and the CD105/CD133 presence or absence

Results obtained from CD105/CD133 FACS and real-time PCR analysis were listed (Table 3) in reference to the appropriate cell line origin.

## Discussion

Previous studies have reported that CD105+ cells may represent a tumor-initiating stem cell population in RCC [13, 42]. However, due to the low abundance of this subpopulation in total tumor samples, the number of studies on CD105+ cells in RCC is limited and they are mostly based on primary cultures and nephrectomy specimens [36]. We aimed to set a feasible laboratory model of CD105+ RCC-CSCs derived from established renal cancer cell lines. We showed that selected cell lines comprised a variable number of CD105+ cells—referred as to CD105high or CD105low cell lines—and that this characteristic was independent of the cell culture source in terms of RCC grade or stage, including primary- and metastatic tumor derived cell lines. Surprisingly, primary pRCC tumor cells (HKCSCs) isolated and cultured in stem cell media were not enriched in CD105+ cells. A significant subpopulation of CD105+ cells (>8% total culture) was detected in the ASE cell line derived from a prenatal kidney. This suggests that normal kidney stem cells may also be CD105+, especially as stemness-linked TFs were expressed in those cells, which could confirm the characteristics of a kidney side population (SP) as previously reported [43].

We have shown that established pRCC cell lines—Caki-2 and ACHN—differed both in the number of CD105+ cells and the stem-like phenotype expression. The CD105low ACHN cell line expressed abundant *oct-4* TF but showed an increased ability for stem-like behavior in comparison to the CD105high Caki-2 cell line, including 3D growth in sphere-promoting conditions and clonogenicity. This is in accordance with research on melanoma cell lines, where no correlation between CD105 expression and in vitro invasiveness was observed [44]. Even though SP and CD105+ cells are considered as stem-like cells in RCC, the ACHN cell line must be considered in general to harbor a high stemness phenotype. High stemness of ACHN was previously described as high self-renewal capacity measured by the ability to grow as spheres when cultured under serum-free, attachment-free stem cell culture conditions [45]. Moreover, it has been previously shown that there is no significant difference in the cell proliferative ability and clonogenicity between the SP and other cells in the ACHN cell line as both populations were highly aggressive. Nevertheless SP cells in ACHN had a significantly higher sphere forming ability than non-SP cells [17], and CSCs were also isolated from the ACHN cell line based on high ALDH expression [46, 47]. Therefore, it could be concluded that CD105+, ALDH-high and SP cells found in the ACHN cell line represent CSCs. However, the ACHN cell line is, in general, highly aggressive and is challenging the CSCs research model because of endogenous mesenchymal phenotype [48] with a higher expression of N-cadherin and vimentin [49].

CD105, a component of the TGFβ receptor complex, is a glycoprotein most often expressed by endothelial cells. It is associated with angiogenesis and tumor neovascularization, and has been shown as an independent marker of a favorable prognosis in RCC. High expression of this protein negatively correlates with the nuclear grade and tumor stage of ccRCC [50]. Surprisingly, an inverse relation seems to characterize pRCC as higher CD105 expression correlated insignificantly with a poor prognosis. At the same time, 39% of pRCCs were negative for CD105 compared to 25% ccRCC. However, as shown by Dubinski et al. [51], in ccRCC, CD105 overexpression was prognostic of a poor outcome. Later, Saroufim et al. [52] revealed that CD105 expression must be distinguished between endothelial or tumor cell expression and that it has either a negative or a positive correlation with the prognosis, respectively. However, this analysis was limited to ccRCC; no information on tumoral CD105 expression in pRCC has been reported until now. In general, pRCC studies are underrepresented as this type of renal cancer is less frequently diagnosed. In our study, CD105 expression in pRCC cell lines negatively correlated with invasiveness in vitro. However, the differences in stem-like features observed in our studies may be correlated more with the RCC stage (metastatic vs. primary) than with an abundance of CD105+ cells in culture.

Although the usability of CD133 as a general CSCs marker is controversial [53, 54], in kidney-focused research, the CD133+ cell population has been identified as resident human renal progenitor cells of an adult normal kidney [40]. CD133+ cells isolated from primary tumors (nephrectomy specimens) of RCC were shown to promote tumor vascularization and neoangiogenesis in the nude mice model [37]. In another trial, CD133+/CD24+/CTR2+ cells were tumorigenic and indicated as RCC CSCs/TICs [55], as well as CD44+/CD133+/CXCR4+ [49] and CD44+/CD105+/CD133+/CD90+ cells [56]. In our study, CD133 was expressed at low levels in established RCC cell lines, which is in accordance with previous data [38, 57]. However, the protein was abundant in healthy kidney ASE cells, which was probably due to their prenatal origin and low differentiation state [58].

CSC (tumor initiating) subpopulations were previously effectively isolated from established cell lines including RCC, mostly with the side-population isolation approach (Rho-negative, Hoehst-negative, ALDH-positive) [17, 59, 60]. In particular, the SP-cells subpopulation isolated with Hoechst 33342 dye from ACHN and RENCA cells were 2.6 and 18%, respectively [15]. SP was later isolated from 769-P, 786-O, OS-RC-2, SN12C, and SKRC39 cell lines [22, 61]. However, currently, there are no reports on the presence of CD105+/CD133+ subpopulations in pRCC cell lines. We have demonstrated the presence of CD105+ cells in established RCC cell lines, including 786-O, SMKT-R2, SMKT-R3, 769-P, Caki-1, Caki-2, ACHN, and RCC6 [62, 63]. In this study, we have shown that the level of CD105+ cells in pRCC is not correlated to the stem phenotype of cells, which may question the usefulness of the single surface marker (CD105) isolation approach to identify CSCs subpopulation in RCC in vitro cell culture model.

The next aim of the study was to unravel the role of hypoxia in pRCC invasiveness and the promotion of stem-like features and marker expression. It is well described that low O_2_ tension develops in tumors in vivo [64, 65] and promotes a CSC phenotype [66]. Our study revealed that selected pRCC cell lines exposed to hypoxia in vitro increased the expression of stem-related TFs (*oct-4* and *sox2*). TF expression was observed both in ACHN cells where growth was suppressed by low O_2_, and in Caki-2 that proliferate more extensively in hypoxia. The above mentioned TFs are known to be up-regulated by HIF2 [67]; however, in tested cells, only ACHN (but not the Caki-2 line) increased *hif2* expression simultaneously with stem-related TFs in hypoxia. This is in accordance with pRCC showing lower *hif2* expression than ccRCC [68]. As a result, HIF2 (and HIF1) may be of secondary importance in CSCs induction in pRCC and other signaling pathways may be responsible for Sox and Oct induction, in particular WNT and Cdx1 [56, 69, 70]. Nevertheless, the up-regulation of stem cell TFs by hypoxia was not correlated with a stem-like phenotype: clonogenicity, and high aggregation properties as expected [71]. Cancer cell migration potential suggests the ability of cells to metastasize^111^. Cancer cell movement in the wound healing assay corresponds to the cell’s ability to exit a primary tumor, to penetrate into blood vessels, and to finally spread in vivo. In many cancer types, it has been proven that internal tumor hypoxia induces cancer migration potential both in vivo [72] and in vitro [73, 74], although our results show the opposite. Regardless of the CD105 or CD133 level, all analyzed cancer cell lines showed reduced migration potential in hypoxia. This phenomenon may be the result of an alternative hypoxia-response mechanism in analyzed pRCC cell lines compared to previous studies. Another pivotal reason is the inability to separately measure two mechanisms—migration and proliferation—in a wound healing assay. In the ACHN cell line of high stemness, we have shown a reduced rate of proliferation under hypoxia with G2/M arrest. However, a stem-like phenotype of these cells was not upregulated by low O_2_. On the other hand, Caki-2 cells, which proliferate more rapidly in hypoxia, simultaneously exerted more stem-like features and grew in large spheres, but not in semi-soft agar while remaining in aggregates in a hanging drop culture. Cumulatively, in vitro assays performed to test cell line aggressiveness and tumorigenicity [75] suggest that CD105high Caki-2 cells express a less aggressive phenotype, but survive better in hypoxia. Moreover, CD105 expression is usually up-regulated by low O_2_ tension, promotes vascularization, and protects cells from hypoxia-induced apoptosis [76]. However, in our study, CD105high cells (Caki-2) proliferated faster in hypoxic conditions while CD105 expression was reduced by hypoxia. The different reactions of Caki-2 and ACHN cells to hypoxia was not dependent on p53 [77] nor on *vhl* status [78], as both cell lines were the *tp53/vhl* wild-type.

## Conclusions

We did not observe a positive correlation of CD105 level and colony formation and clonogenicity in tested ACHN and Caki-2 cell lines, neither in normoxic nor hypoxic conditions. The Caki-2 pRCC cell line, characterized as CD105high, had weaker stem features when compared to the CD105low ACHN cell line with a more aggressive phenotype. The differences in stem potential of those cells describe the characteristics of primary and metastatic cells rather than the contribution of CD105+. Also, in contrast to other studies, we have shown that in the case of pRCC, hypoxic conditions in vitro diminish the stem-like properties of cell lines [79–85].

## Abbreviations

ALDH: Aldehyde dehydrogenase
ccRCC: Clear cell renal cell carcinoma
CD105: Endoglin
CD133: Prominin-1
CD24: Cluster of differentiation 24
CD44: Indian blood group
CD90: Cluster of differentiation 90
chRCC: Chromophobe renal cell carcinoma
CSC: Cancer stem cells
CTR2: Cooper uptake 2
CXCR4: C-X-C chemokine receptor type 4
HIF1: Hypoxia-inducible factor 1
HIF2: Hypoxia-inducible factor 2
HKCSC: Human kidney cancer stem cells
Oct4: Octamer-binding transcription factor 4
PPIA: Peptidylprolyl isomerase A
pRCC: Papillary renal cell carcinoma
RCC: Renal cell carcinoma
Sox2: SRY (sex determining region Y)-box
TF: Transcription factor
TGFβ: Transforming growth factor beta
